# The Long-term Effect of Superficial Bladder Neck Incision on Ejaculation and Incontinence in Boys with Primary and Secondary Bladder Neck Obstruction

**DOI:** 10.3389/fped.2017.00152

**Published:** 2017-07-13

**Authors:** Pauline M. L. Hennus, Esther Hoenjet, Jan H. Kieft, Tom P. V. M. de Jong, Laetitia M. O. de Kort

**Affiliations:** ^1^Department of Urology, University Medical Center Utrecht, Utrecht, Netherlands; ^2^Department of Pediatric Urology, University Children’s Hospitals UMC Utrecht and AMC Amsterdam, Utrecht, Netherlands

**Keywords:** bladder neck incision, bladder neck obstruction, boys, long-term follow-up, posterior urethral valves, retrograde ejaculation

## Abstract

**Objective:**

Superficial bladder neck incision (SBNI) is controversial at young age, with retrograde ejaculation after puberty as main concern. The aim of the study is to investigate the long-term effect of SBNI on ejaculation and incontinence in boys with primary and secondary bladder neck obstruction (BNO).

**Materials and methods:**

In boys with infravesical obstruction, SBNI was performed in case of a persistent BNO after earlier desobstruction or in case of primary severely obstructive bladder neck. SBNI was performed with a diathermy hook, always superficially (2–3 mm) and unilaterally at 7 O’clock. Males that had SBNI during childhood after posterior urethral valve incision or relief of other obstruction between 1986 and 2003 were included. Evaluation was done by *International Continence Society male sex questionnaire, International Prostate Symptom Score*, developmental *International Consultation Modular Questionnaire on Urinary Incontinence*, frequency volume chart, and uroflowmetry.

**Results:**

Of 79 traceable patients, 40 (50.6%) participated. Of these, 37 (92.5%) completed all questionnaires and 28 (70%) performed uroflowmetry. Median age at SBNI was 4.7 years [interquartile range (IQR) 0.6–8.5] and was 19.6 years (IQR 17.3–20.9) at follow-up. All men had antegrade ejaculation, 4/37 (10.8%) reported possibly reduced ejaculatory volume. Eight (22%) had moderate lower urinary tract symptoms and two (5.4%) had moderate incontinence. Median maximum flow rate was 30.1 mL/s (IQR 24.4–42.6).

**Conclusion:**

SBNI in boys with severe infravesical obstruction can be done safely with preservation of antegrade ejaculation and no additional lower urinary tract dysfunction.

## Introduction

Bladder neck obstruction (BNO) is a condition described as an insufficient opening of the bladder neck during voiding due to a functional or anatomic narrowing of the bladder neck and was first described by Marion in 1933 (1). The underlying cause of primary BNO remains unclear. In boys with posterior urethral valves (PUV), BNO is often present and caused by detrusor hypertrophy with subsequent narrowing of the bladder neck (2, 3). In most cases of PUV, BNO disappears spontaneously over time after PUV incision due to disappearance of detrusor hypertrophy. Persistent BNO after PUV or other obstruction incision may contribute to lower urinary tract symptoms (LUTS), urinary incontinence (UI), urinary tract infections (UTIs), and vesicoureteral reflux (3–6). Untreated persistent bladder outlet obstruction may lead to progressive symptoms, as well as upper and lower urinary tract decompensation (2, 7).

In the past, persistent BNO in children has been treated by Y-V bladder neck plasty. Others treated BNO by temporary vesicostomies or by alpha blocking pharmaceuticals. Many believe that bladder neck hypertrophy and narrowing is just the consequence of a more distal obstruction and does not act as an obstruction itself. After PUV incision (PUVI), the appearance and function of the bladder neck usually improves (3). In 1973 Turner-Warwick et al. introduced a bladder neck incision (BNI) technique in adults (8). After bilateral BNI in adult men, retrograde ejaculation occurs in 21% (9). Also, unilateral BNI in adults reduced sperm count to 70%, while a limited unilateral BNI seemed to preserve antegrade ejaculation in most men (10, 11). In order to avoid complications, when needed, we perform a unilateral superficial bladder neck incision (SBNI) only at 7 O’clock. It is unknown whether complications after BNI in adults, such as retrograde ejaculation, reduced sperm count, and bladder neck stricture, will also appear after SBNI in early childhood. In particular, retrograde ejaculation is a concern for these boys who might want to become fathers in the future. For this reason, SBNI in boys and young men remains controversial.

The goals of this study were to investigate the presence of antegrade ejaculation on the long term after unilateral SBNI performed during childhood and to evaluate long-term voiding symptoms. Primary investigation tools were questionnaires concerning sexual function and voiding problems.

## Materials and Methods

### Study Population

This observational cross-sectional cohort study was approved by the local ethical committee. Male patients who underwent transurethral treatment for infravesical obstruction at our institution between January 1985 and December 2003 were retrospectively retrieved through the hospital surgical registration database. Patients with neurogenic bladder or congenital anomaly of the penis (hypospadias or epispadias) were excluded. Contact details of patients were obtained. Patients were informed by telephone about the goal and design of the study and asked to complete written informed consent, questionnaires, and a frequency volume chart (FVC). Patients were invited to our outpatient clinic for uroflowmetry. Baseline characteristics were obtained by reviewing medical records.

**Figure 1:**
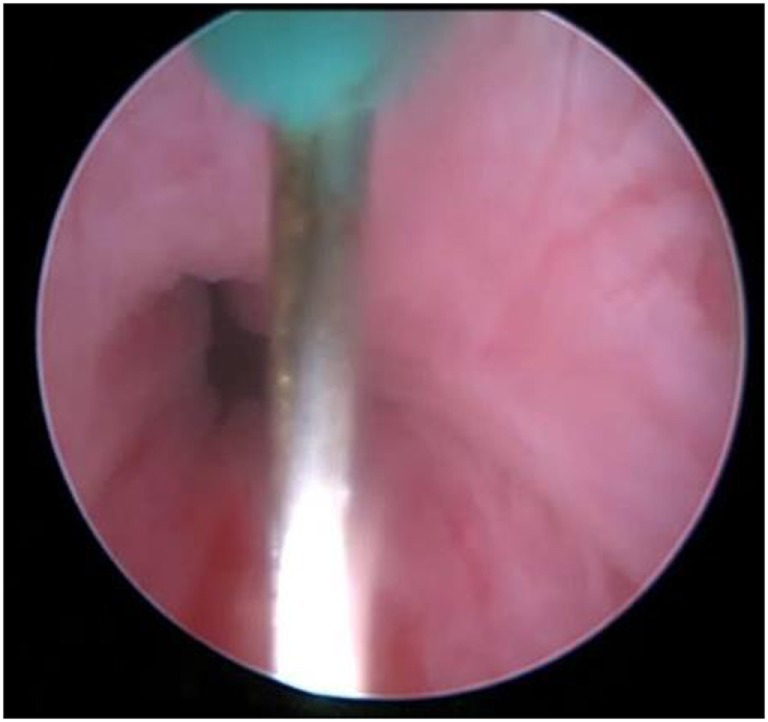
Obstructive bladder neck, proven with urodynamic study.

**Figure 2:**
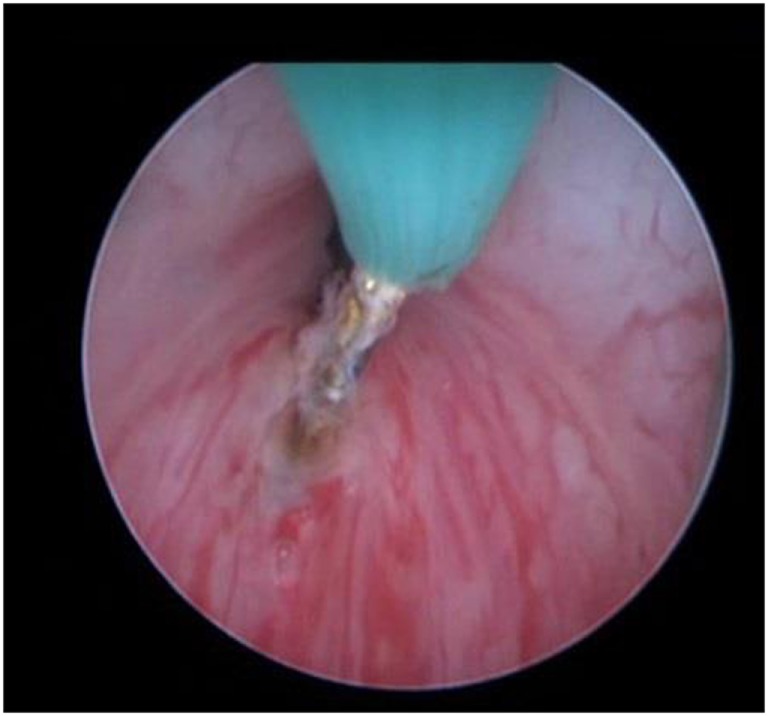
Performing superficial bladder neck incision with hook diathermia.

**Figure 3:**
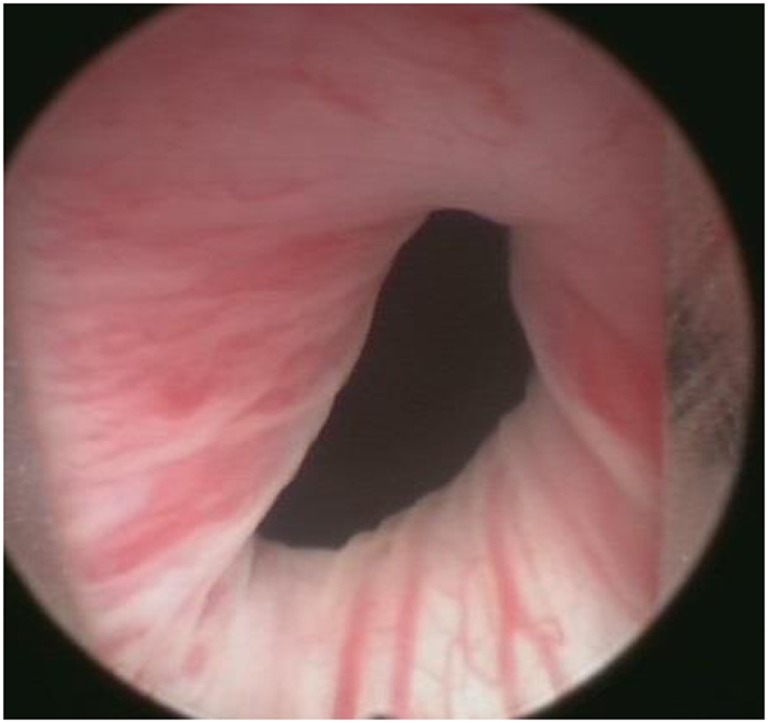
10 months after superficial bladder neck incision at 7 O’clock.

Included patients were 18 years or older at time of study and did have SBNI at early age. Cystoscopy was performed based on complaints very suggestive for obstruction, obstructive flow, or urodynamic study proving obstruction. Primary SBNI was performed when the bladder neck was considered severely obstructive during cystoscopy. SBNI was performed with a diathermy hook, always superficially (2–3 mm) and unilaterally at 7 O’clock. The incision was done to interrupt the obstructive circle. In Figures 1–3 we show examples before and after treatment. Secondary SBNI was performed if BNO did not disappear after endoscopic desobstruction (ED) of PUV or any other obstructive structure. Persistent BNO was confirmed by urodynamic study (Pdet-Q_max_ > 50 cmH_2_O) or voiding cystography (constriction of the bladder neck). Patients have been followed subsequently until confirmation that LUTS had subsided. Patients with a history of PUV, upper urinary tract problems, and a valve bladder were kept under regular controls till after puberty.

### Study Parameter

#### Questionnaires

Questions 3 and 4 of the International Continence Society male sex questionnaire (ICS male sex) were used to evaluate the presence of antegrade ejaculation. The International Prostate Symptom Score (IPSS) and quality of life (IPSS-QoL) and questions 7–12 of the developmental version of the International Consultation on Incontinence Modular Questionnaire—Urinary Incontinence (ICIQ-UI) were also used (12–14).

The IPSS was divided in four categories: “no symptoms” (score 0), “mild symptoms” (score 1–7), “moderate symptoms” (score 8–19), and “severe symptoms” (score 20–35). Individual IPSS question scores were divided in three categories: “never” (score 0), “rarely” (score 1), and “regularly/often” (score 2–5).

Because it was unknown whether SBNI would lead to a specific form of incontinence, the developmental ICIQ-UI questionnaire was used and not the ICIQ-*short form*. The ICIQ-UI questionnaire makes it possible to distinguish between stress, urge, nocturnal, and post-void incontinence, or incontinence for unknown reason. The ICIQ-UI score was divided in four categories: “no symptoms” (score 0), “mild symptoms” (score 1–6), “moderate symptoms” (score 7–15), and “severe symptoms” (score 16–24). Individual ICIQ-UI question scores were divided in three categories: “never” (score 0), “rarely” (score 1), and “regularly/often” (score 2–4). For the content of the questionnaires and additional questions, see the Tables 1–4 and Box 1.

**Table 1:** Baseline characteristics.

Total no. of patients	40	All	27 BNI and PUV	13 BNI no PUV	*p*-Value
Median age at first ED, years (IQR)	2.7	(0.2–7.8)	1.9 (0.1–7.5)	4.9 (0.3–8.3)	0.36
Median age at SBNI, years (IQR)	4.7	(0.6–8.5)	3.2 (0.9–9.4)	4.9 (0.4–8.3)	0.67
Median age at follow-up, years (IQR)	22.5	(20.8–26.2)	22.4 (20.5–26.3)	24.0 (21.3–26.7)	0.30
Median follow-up time, years (IQR)	19.6	(17.3–20.9)	19.6 (18.8–20.7)	19.6 (16.9–21.1)	0.92
Number of endoscopic procedures					0.01
1 time (%)	21	(52.5)	10 (37)	11 (85)	
2 times (%)	11	(27.5)	10 (37)	1 (7.5)	
3 times (%)	4	(10.0)	4 (15)	0	
4 times (%)	4	(10.0)	3 (11)	1 (7.5)	
Endoscopic procedures					
No. PUVI (%)	27	(67.5)	27 (100)	0 (0)	
No. urethrotomia (%)	19	(47.5)	13 (48)	6 (46)	
No. dilatation (%)	11	(27.5)	9 (33)	2 (15)	
No. utricular remnant incision (%)	10	(25)	8 (30)	2 (15)	
No. other ED, with incision (%)	2	(5)	1 (3.7)	1 (7.5)	

*SBNI, superficial bladder neck incision; BNI, bladder neck incision; ED, endoscopic desobstruction; PUV, posterior urethral valve; PUVI, PUV incision; IQR, interquartile range; No., number; VUR, vesicoureteral reflux*.

**Table 2:** Questionnaire results.

Total patients in study (%)	40	(100)	BNI and PUV	BNI no PUV	*p*-value
No. of respondents (%)	37	(92.5)			
**ICS 3—ejaculation**					
No. normal ejaculation (%)	33	(89.2)	22 (92)	11 (85)	
No. ejaculation with reduced amount (%)	4	(10.8)	2 (8)	2 (15)	
No. ejaculation not anymore/never had (%)	0	(0)	0 (0)	0 (0)	
**ICS 4—pain or discomfort during ejaculation**					
No. no pain or discomfort (%)	34	(91.9)	22 (92)	12 (92)	
No. minor pain or discomfort (%)	3	(8.1)	2 (2)	1 (8)	
No. moderate/severe pain or discomfort (%)	0	(0)	0 (0)	0 (0)	
**IPSS**					
Median score (IQR)	4	(1–6)	2.5 (1–6)	5 (3–10)	0.03
Median bother score (IQR)	0	(0–1.5)	0 (0–1)	0 (0–2)	0.67
No. no LUTS (%)	5	(13.5)	5 (21)	0 (0)	
No. mild LUTS (%)	24	(64.9)	15 (62)	9 (69)	
No. moderate LUTS (%)	8	(21.6)	4 (17)	4 (31)	
No. severe LUTS (%)	0	(0)	0 (0)	0 (0)	
**IPSS 8—quality of life**					
No. satisfied (%)	33	(89.2)	21 (59)	12 (92)	
No. equally satisfied/dissatisfied (%)	2	(5.4)	2 (29)	0 (0)	
No. dissatisfied (%)	2	(5.4)	1 (8)	1 (8)	
**ICIQ-UI**					
Median score (IQR)	1	(0–2.5)	0 (0–1)	2 (1–3.5)	0.01
Median bother score (IQR)	0	(0–1)	0 (0–0)	0 (0–1)	0.17
No. no UI (%)	16	(43.2)	14 (58)	2 (15)	
No. mild UI (%)	19	(51.4)	8 (34)	11 (85)	
No. moderate UI (%)	2	(5.4)	2 (8)	0 (0)	
No. severe UI	0	(0)	0 (0)	0 (0)	

*ICIQ-UI, International Consultation on Incontinence Modular Questionnaire—Urinary Incontinence; ICS, International Continence Society; IPSS, International Prostate Symptom Score; LUTS, lower urinary tract symptoms; IQR, interquartile range; No., number; UI, urinary incontinence; PUV, posterior urethral valve; BNI, bladder neck incision*.

**Table 3:** Questionnaire results IPSS and ICIQ-UI.

IPSS	Never	<1/5 of time	<1/2 of time	>1/2 of time

	*N* (%)	*N* (%)	*N* (%)	*N* (%)
Incomplete emptying	21 (57)	9 (24)	5 (13)	2 (6)
Frequency	11 (30)	16 (43)	7 (19)	3 (8)
Intermittency	24 (65)	9 (24)	3 (8)	1 (3)
Urgency	26 (70)	8 (22)	2 (5)	1 (3)
Weak stream	21 (57)	13 (35)	1 (3)	2 (5)
Straining	25 (68)	8 (22)	1 (3)	3 (8)
Nocturia	25 (68)	10 (27)*	1 (3)**	1 (3)***

**ICIQ-UI**	**Never**	**Rarely**	**Regularly**	**Mostly**

Urgency	24 (65)	9 (24)	4 (11)	0 (0)
Urge incontinence	31 (84)	4 (11)	2 (5)	0 (0)
Stress incontinence	37 (100)	0 (0)	0 (0)	0 (0)
Incontinence eci	33 (89)	3 (8)	1 (3)	0 (0)
Nocturnal incontinence	32 (87)	5 (13)	0 (0)	0 (0)
Post-void dribbling	22 (60)	11 (30)	3 (8)	0 (0)

*IPSS, International Prostate Symptom Score; ICIQ-UI, International Consultation on Incontinence Modular Questionnaire—Urinary Incontinence*.

*1 time nocturia, **2 times nocturia, ***3 or more times nocturia.

**Table 4:** Uroflowmetry results.

Uroflowmetry	No. respondents (%)	Median	(IQR)
Total respondents	40 (100)		
Voiding time (s)	28 (70)	25.5	(17.3–35.5)
Flow time (s)	28 (70)	26	(16.3–38.3)
Time to peak (s)	28 (70)	9	(6–11)
Q_max_ (mL/s)	28 (70)	30.1	(24.4–42.6)
Average flow rate (mL/s)	28 (70)	17.4	(11.6–21.8)
Total voided volume (mL)	28 (70)	467.5	(285.3–582.3)
PVR (mL)	28 (70)	57.5	(23.8–100)

*IQR, interquartile range; No., number; PVR, post-void residual; Q_max_, maximum flow rate*.

**Box 1:** Questionnaires.

**ICS *male sex* questions**	**ICS *male sex* bother questions**

1a. Do you have an ejaculation of semen?	b. How much of a problem is that for you?
**Score:** *0* = *yes, normal, 1* = *yes, reduced, 2* = *yes, strongly reduced, 3* = *not anymore, 4* = *never had*	
2a. Do you have pain or discomfort during ejaculation?	
**Score:** *0* = *no, 1* = *yes, a little pain/discomfort, 2* = *yes, rather pain/discomfort, 3* = *yes, severe pain/discomfort, 4* = *not applicable*	**Score:** *0* = *no problem, 1* = *a little problem, 2* = *a serious problem, 3* = *a major problem*

**ICIQ-UI questions**	**ICIQ-UI bother questions**

1a. Do you have a sudden need to rush to the toilet to urinate?	b. How much are you bothered?
2a. Does urine leak before you can get to the toilet?	
3a. Does urine leak when you cough or sneeze?	
4a. Do you ever leak for no obvious reason and without feeling that you want to go?	
5a. Do you leak urine when you are asleep?	
6a. How often have you had a slight wetting of your pants a few minutes after you had finished urinating and had dressed yourself?	
**Score:** *0* = *never, 1* = *occasionally, 2* = *sometimes, 3* = *most of the time, 4* = *all of the time*	**Score:** *0* = *not at all, 1* = *slightly, 2* = *moderately, 3* = *greatly*

***Total score*:** *0*–*24*	***Total score*:** *0*–*18*

#### Measurements

Uroflowmetry using an UroDyn^®^ 1000 was performed. Post-void residual (PVR) was measured using a BladderScan^®^. Obtained parameters were voiding time, flow time, maximum flow rate (Q_max_), time to Q_max_, average flow rate, voided volume, and flow pattern. Measurements with a voided volume ≤100 mL, or not representative flow according to the patient, were excluded from analysis. The Pernkopf nomogram was used for interpretation to plot Q_max_ versus voided volume (15). The ICS nomenclature was used to describe the flow curves. A PVR of ≥100 mL was defined as significant in these adult young men.

### Statistical Analysis

Descriptive statistics were used. Data are presented as median with interquartile range (IQR), frequencies, and percentages. IPSS and ICIQ-UI scores were categorized. Data were analyzed using IBM-SPSS^®^ version 20.0 for Windows with chi-square test and Mann–Whitney *U* test.

## Results

### Study Population

#### General

During the study period, 106 patients underwent SBNI; 79 were traceable, 42 patients returned questionnaires or performed uroflowmetry. Two of them did not sign informed consent for unknown reasons. Of the 40 included patients, 37 (92.5%) fully completed the questionnaires and 30 (75%) performed uroflowmetry. Two uroflowmetry measurements were excluded: one because of a voided volume <100 mL and another because it was not representative.

#### Baseline

Table 1 presents the baseline characteristics for the entire group and also for the patients with PUV and patients without PUV. Median age at first ED was 2.7 years (IQR 0.2–7.8), and at the SBNI, the median age was 4.7 years (IQR 0.6–8.5). In 27 patients a primary BNI was performed. Median follow-up time since first ED was 19.6 years (IQR 17.3–20.9). Median age at follow-up was 22.5 years (IQR 20.8–26.2). Most patients underwent multiple interventions: 27 patients had PUVI, 19 internal urethrotomy for distal urethral stenosis, 11 urethral dilatation, and 10 incision of an utricular remnant. Thirteen patients had BNI combined with another desobstruction than PUV. Six patients were treated with SBNI alone. Age at first treatment was younger in the BNI and PUV group; 1.9 versus 4.9 years, *p* = 0.3. Of patients with PUV, 63% needed re-intervention versus 15% in the group without PUV, *p* < 0.001.

### Questionnaires

Tables 2 and 3 present the results of the questionnaires for the whole group and for the patients with or without PUV.

#### ICS Male Sex

None of the patients experienced antegrade ejaculation. Four patients (10.8%) had the impression of a reduced ejaculatory volume and three (8.1%) had minor discomfort during ejaculation. We found no significant difference between patients treated with BNI and PUV and patients with BNI without treatment of PUV, *p* = 0.51.

#### International Prostate Symptom Score

The median IPSS was 4 (IQR 1–6) and IPSS-QoL was 1 (IQR 0–1). IPSS correlated with QoL with a coefficient of 0.46 (*p* = 0.005). Of the 8 patients that reported moderate symptoms, 4 had PVR >100 mL versus 20 patients with PVR who reported no or mild symptoms based on IPSS. No correlation was found between IPSS symptom score and persistence of PVR, *p* = 0.32. None of the patients in both groups reported severe symptoms. Table 3 shows the results of the individual IPSS questions. We also compared patients with and without treatment for PUV in combination with BNI. Patients with BNI and PUV scored median IPSS of 2.5, while patients with BNI and without PUV scored a median IPSS of 5, *p* = 0.03.

#### International Consultation on Incontinence Modular Questionnaire—Urinary Incontinence

The median ICIQ-UI score was 1 (IQR 0–2.5) with an ICIQ-UI bother score of 0 (IQR 0–1). ICIQ-UI scores correlated with the bother scores with a coefficient of 0.72 (*p* < 0.0001). Patients without PUV reported to have more often mild UI (85%) than patients also treated for PUV (34%), *p* = 0.01. The two patients who reported moderate symptoms also had a PVR >100 mL. None of the patients reported stress or nocturnal incontinence. Table 3 shows the results of the individual ICIQ-UI questions.

### Uroflowmetry

**Figure 4:**
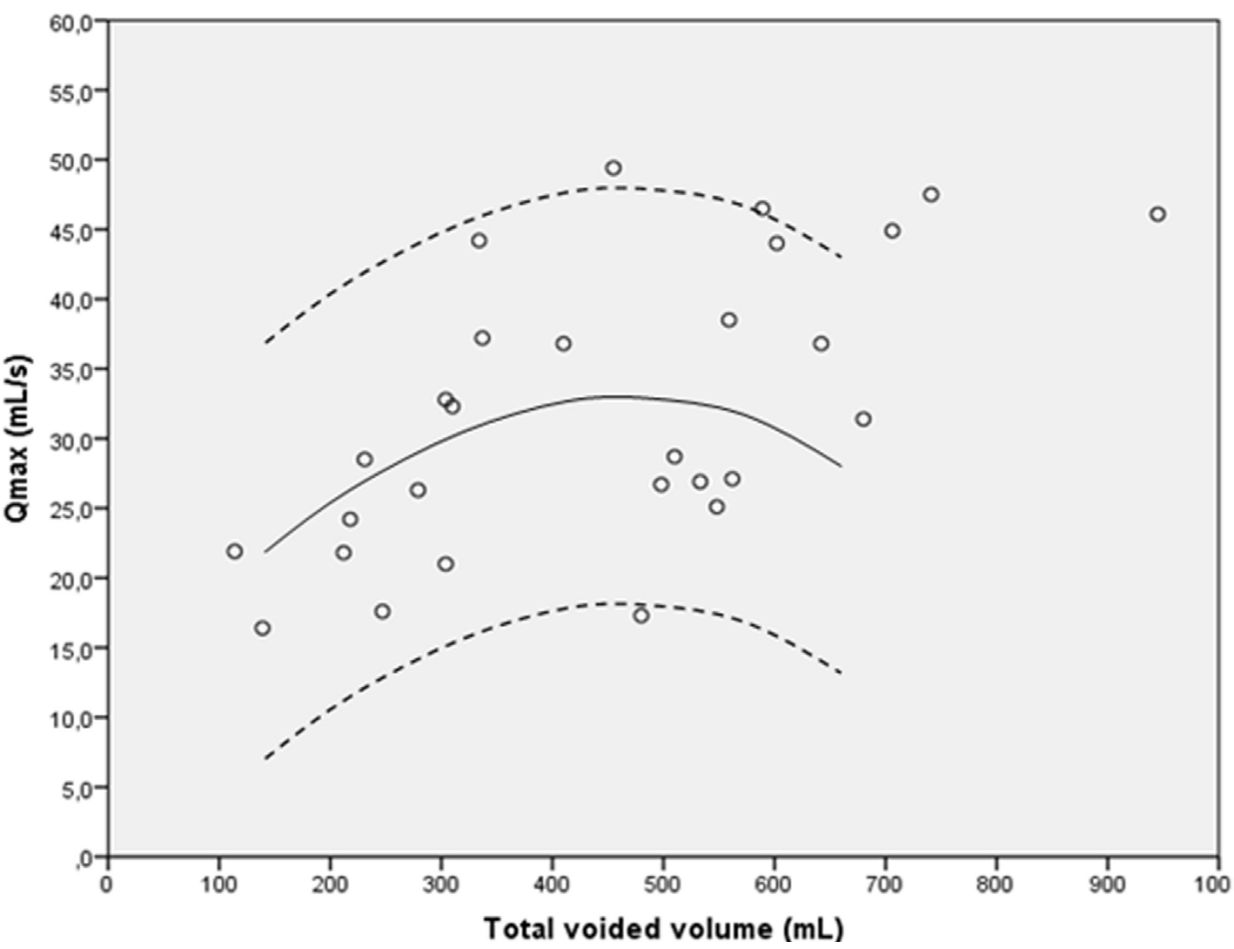
Pernkopf nomogram for bladder neck incision ≤18 years (*n* = 28). The solid line is the 50th percentile, and the dotted lines represent the 5th and 95th percentile.

Table 4 presents the uroflowmetry results. A bell shaped pattern was seen in 21 (75%) of patients, staccato pattern in 6 (21%), and a plateau-shaped pattern was seen in 1 patient (3.6%). One uroflowmetry result (3.6%) was under the 5th percentile of the Pernkopf nomogram, and five (17.9%) were above the 95th percentile. Figure 4 presents the Pernkopf nomogram.

### Further Follow-up

Five patients were advised to revisit the outpatient clinic based on complaints or uroflowmetry results. Three patients showed up for further analysis. The first patient had a high voided volume (945 mL) and large PVR (230 mL) with a normal flow pattern; timed voiding was advised. The second patient had a staccato flow pattern with a significant PVR; he started with clean intermittent catheterization. The third had six UTIs in the past year; a normal flow pattern, normal kidneys on ultrasound, and normal cystoscopy results were found. No urethral strictures or upper tract changes were found.

## Discussion

In this study, we investigate long-term preservation of antegrade ejaculation after BNI in boys. Nearly 20 years after surgery, all of the 40 men experienced antegrade ejaculation, while 4 men had the impression of reduced ejaculatory volume. In this study we did not perform sperm analysis; this would have been useful to quantify our results. We chose not to include sperm analysis because we were afraid that this young adult group of men would refrain from participating. Prevalence of retrograde ejaculation in the general adolescent population is unknown. In patients attending fertility clinics, the prevalence ranges from 0.3 to 2% (16). In other studies investigating retrograde ejaculation after unilateral BNI in adult patients, prevalence ranges from 0 to 16% (17, 18). In a study performed by Taskinen et al. investigating sexual function after PUVI (regardless of BNI), similar prevalence of erectile dysfunction and similar paternity rates are reported compared to men without PUVI. Erectile function and paternity rates were satisfactory in spite of chronic renal failure (19). In a study by Kajbafzadeh et al., no retrograde ejaculation was found after BNI at young age and no reduced sperm volume with normal sperm count in one out of six patients (20). Keihani et al. found similar results in 18 young adult men after concurrent treatment of PUV and BNI (21). The fact that a unilateral superficial BNI was done, only incising the obstructive ring, and thereby avoiding damage to the internal sphincter may contribute to the absence of retrograde ejaculation.

The goal of BNI is to achieve bladder outlet desobstruction in case of persistent BNO after PUVI or in case of severe primary BNO. The beneficial effect of BNI is difficult to determine, because no controlled studies exist. In one semi-randomized study (type of procedure selected by the parents) unilateral BNI seemed urodynamically favorable over PUVI alone (20).

We found on the long term at adult age that 13.5% of patients had no LUTS, 64.9% had mild symptoms, and 21.6% had moderate symptoms. An earlier study in 151 healthy male medical students of the same age as our population revealed moderate LUTS in 7% (22). In a long-term follow-up study using the IPSS in patients who underwent PUVI before age 3 years, LUTS were found in 7/24 children (29.1%) (23). In another long-term follow-up study in young adults treated for mild urethral obstruction in childhood, few micturition symptoms were found, not different from a reference group (24). When comparing our results with a multinational population-based (EPIC) survey in men aged 18–30 years (*n* = 1,370), especially the symptom *frequency* was more prevalent in our study population (27 versus 4%) (25). Unfortunately, the definition of presence or absence of symptoms was unclear in the EPIC study. Tikkinen et al. compared LUTS in men (age 18–57 years) who had PUVI during childhood to the general population (26). They found a twofold increase in prevalence of most LUTS in those with PUVI.

In the present study, two patients (5.4%) reported moderate UI; this is higher than the prevalence found in the EPIC study (2.4%) and in a national survey in the USA in men aged 20–34 years (0.7%) (25, 27). However, in the latter study a different questionnaire than ICIQ-UI was used. Tikkinen et al. found a threefold higher prevalence of urgency incontinence and stress incontinence in patients treated with PUVI compared to the general population (26). In a previous long-term follow-up study, urgency incontinence and post-micturition incontinence were reported in 2.4 and 8.5% of men treated for mild PUV in childhood (24).

Besides showing results for the group as a whole, we also showed results for boys treated for BNI, PUV, and sometimes other urethral obstruction versus boys treated for BNI, sometimes with other urethral obstruction but without PUV. The patients not treated for PUV reported significant higher IPSS and ICIQ-UI scores, although scores were still in mild complaints rates. BNO without PUV may have another cause than secondary BNO due to PUV. In patients with PUV, the BNO is thought to be a result of detrusor hypertrophy caused by obstruction. The different cause of primary BNO may be responsible for other long-term outcomes.

We did not find significant changes between the two groups, but both groups were small. Furthermore, as in both groups in a large part of the patients also other urethral obstructions were present, we cannot conclude that the presence of PUV influences outcome based on this study.

Uroflowmetry results were in the normal range and very similar to the mean values of healthy male students of the same age (22). Similar results were found in a previous study in men after PUVI as a child (23). A plateau-shaped curve (possibly indicative of urethral stricturing) was seen in one patient (3.6%). However, in healthy male students a plateau-shaped curve was found in 5% (23). In our patients with abnormal flow pattern, urethral stricture could not be confirmed. No clear explanation can be found for these anomalous uroflowmetry results. As only single measurements were done, repeated uroflowmetry might have revealed other results.

Overall, long-term effect on micturition in men with childhood desobstruction with or without BNI as a child has been studied relatively well. Regarding LUTS, outcomes vary widely, varying between no difference to twice as much symptoms as in the general population, with irritative LUTS being most prominent. Incontinence seems more prevalent than in the general population, and uroflowmetry is not clearly different from controls. However, the question whether additional or primary SBNI is beneficial and recommendable is still unclear and has to be determined in a large randomized study.

A limitation of the present study is the relatively low response rate (37.7%); outdated contact details made it difficult to contact selected patients. Also, among the contacted patients, some refused to participate and some did not respond. The reason for refusal varied from: no time, no complaints, or unpleasant associations with hospitals. This may have resulted in some selection bias in the way that there might be an overrepresentation of patients with complaints. The response rate compares with a large meta-analysis by Shih and Fan showing an average response rate for young adults of 45% for mail surveys and a response rate of 60% of traceable patients as found by Larcombe et al. (28, 29).

Other shortcomings are the lack of randomization in determining whether or not SBNI was done, and the fact that for primary SBNI assessment of the bladder neck was done subjectively by the pediatric urologist. In cases of secondary SBNI, this was performed after urodynamic proof of persistent infravesical obstruction after earlier urethral desobstruction. The questionnaires used were validated, whereas the additional questions were not. Because ejaculation volume is difficult to objectify, interpretation of the reported data should be done with caution. A limitation of the ICIQ-UI is that the amount of urine loss is not obtained; it questions whether there is leakage but does not distinguish between urine droplets and large volumes. We did not do repeated cystoscopy as a routine, because in pediatric patients general anesthesia is needed. In selected cases who had cystoscopy after SBNI, a normal appearance of the bladder neck was seen.

## Conclusion

Bladder neck incision is sometimes indicated and can be done safely. There seems no negative long-term effect on antegrade ejaculation and incontinence.

## Ethics Statement

This study was carried out in accordance with the recommendations of the medical ethic committee of University Medical Center Utrecht with written informed consent from all subjects. All subjects gave written informed consent in accordance with the declaration of Helsinki.

## Author Contributions

EH contributed to the collection of patient information and follow-up data, processing data, and writing the manuscript. PH contributed to data collection, assisted with interpreting results as well as writing and changes to the manuscript. JK worked on the collection of patient information and follow-up data and contributed to manuscript. TJ and LK contributed to study outline and writing the manuscript.

## Conflict of Interest Statement

None of the authors has any conflict of interest to report. No external funding has been used for this study. The study has been approved by the local ethical committee.

## Abbreviations

SBNI, superficial bladder neck incision; BNO, bladder neck obstruction; ED, endoscopic desobstruction; FVC, frequency volume chart; ICIQ-UI, International Consultation on Incontinence Modular Questionnaire—Urinary Incontinence; ICS *male sex*, International Continence Society *male sex*; IPSS, International Prostate Symptom Score; IQR, interquartile range; LUTS, lower urinary tract symptoms; PUV, posterior urethral valve; PUVI, posterior urethral valve incision; PVR, post-void residual; QoL, quality of life; Q_max_, maximum flow rate; UI, urinary incontinence; UTIs, urinary tract infections; VUR, vesicoureteral reflux.
